# Ischemic stroke related to HIV and SARS-COV-2 co-infection: a case report

**DOI:** 10.1590/0037-8682-0692-2020

**Published:** 2020-11-25

**Authors:** Paula Bonates Bessa, Andreza Karoline Barros Brito, Flávio Ribeiro Pereira, Sildomar Queiroz e Silva, Taynná Vernalha Rocha Almeida, André Patrício de Almeida

**Affiliations:** 1 Fundação de Medicina Tropical Dr. Heitor Vieira Dourado, Manaus, AM, Brasil.; 2 Fundação Centro de Controle de Oncologia do Estado do Amazonas, Manaus, AM, Brasil.; 3 Universidade Federal do Amazonas, Faculdade de Medicina, Programa de Pós-graduação em Ciências da Saúde, Manaus, AM, Brasil.

**Keywords:** HIV, SARS-CoV-2 Infection, Ischemic Stroke, Covid-19

## Abstract

A 56-year-old male with human immunodeficiency virus required hospitalization due to the onset of both dyspnea and asthenia. A computed tomography of the chest exam showed the radiological pattern of coronavirus SARS-CoV-2 pulmonary involvement. Based on immunochromatographic analysis, the patient evolved as a reagent for immunoglobulin M (IgM) and immunoglobulin G (IgG) anti-SARS-CoV-2 antibodies. The individual developed complete hemiparesis with a predominance in the right arm and conduction aphasia. T1-weighted magnetic resonance sequence of the brain showed an area of hypointensity with a high intrinsic cortical signal and hyperintensity in the T2-sequence. A Doppler velocimetric examination showed total/critical sub occlusion, suggesting an ischemic stroke.

## INTRODUCTION

As its name implies, severe acute respiratory syndrome coronavirus 2 (SARS-CoV-2) infection commonly leads to respiratory symptoms. However, other symptoms have also been reported, including stroke, Guillain-Barré syndrome, transverse myelitis, and encephalitis. Notably, neurological manifestations may appear prior to respiratory symptoms, even among patients with mild symptoms and without a relationship between symptom severity and the involvement of these systems^1^. In this context, we present a case report of a patient coinfected with pneumonia resulting from SARS-CoV-2 and the human immunodeficiency virus (HIV), specifically regarding the clinical evolution of acute ischemic syndrome after the resolution of the patient’s respiratory conditions.

## CASE REPORT

The patient was a male aged 56 years with comorbidities of diabetes and HIV. This individual was clinically and laboratory-compensated. The patient was using tenofovir, lamivudine, and efavirenz. His TCD4 lymphocyte count was measured at 1,163 cells, with a viral load that remained undetectable for a long period of time. However, hospitalization was required due to the onset of both dyspnea and asthenia. While a laboratory investigation via reverse transcription polymerase chain reaction (RT-PCR) showed negative results for SARS-CoV-2 infection, a computed tomography (CT) examination of the chest showed a radiological pattern typically reported following the pulmonary involvement of SARS-CoV-2. Based on immunochromatographic analysis, the patient evolved as a reagent for immunoglobulin M (IgM) and immunoglobulin G (IgG) anti-SARS-CoV-2 antibodies. The patient was clinically compensated for respiratory infection, initially treated with ceftriaxone and clarithromycin for 3 days, and did not require other therapeutic measures to control the respiratory condition. Two days after hospital discharge (around 20 days after the beginning of respiratory symptoms) the patient developed complete hemiparesis with a predominance in the right arm and conduction aphasia. At that time, the patient scored 11 on the National Institutes of Health Stroke Scale. An electrocardiogram showed sinus tachycardia, while a Doppler echocardiogram showed results within the normal range. Lumbar puncture was performed for cerebrospinal fluid (CS) analysis and was not compatible with a central nervous system infection or acute disseminated encephalomyelitis. 

**FIGURE 1:**
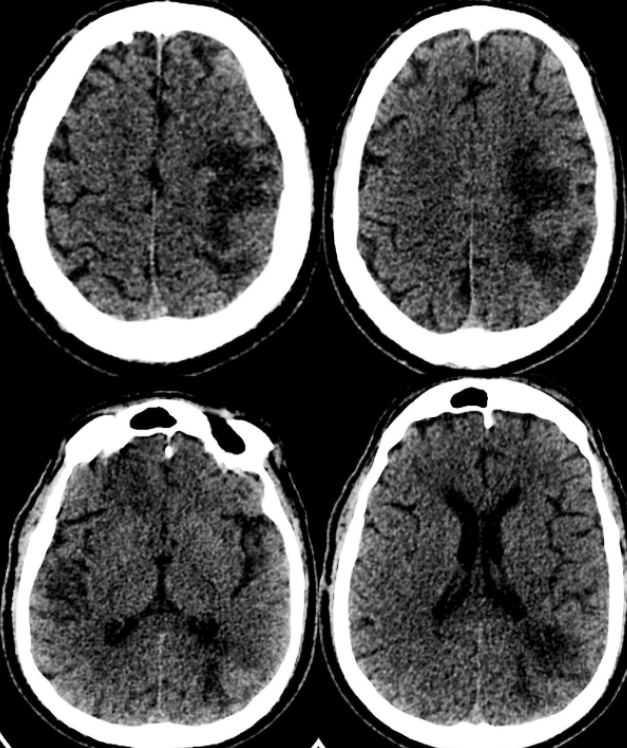
Computed Tomography of the skull without contrast showing loss of differentiation between the white-gray substance, hypoattenuation of deep nuclei, and an extensive area of the vascular territory of the left middle cerebral artery.

Imaging examinations were also conducted, with CT of the skull showing a loss of differentiation between the white-gray substance, hypoattenuation of deep nuclei, and an extensive area of vascular territory in the left middle cerebral artery (Figure 1). 

**FIGURE 2:**
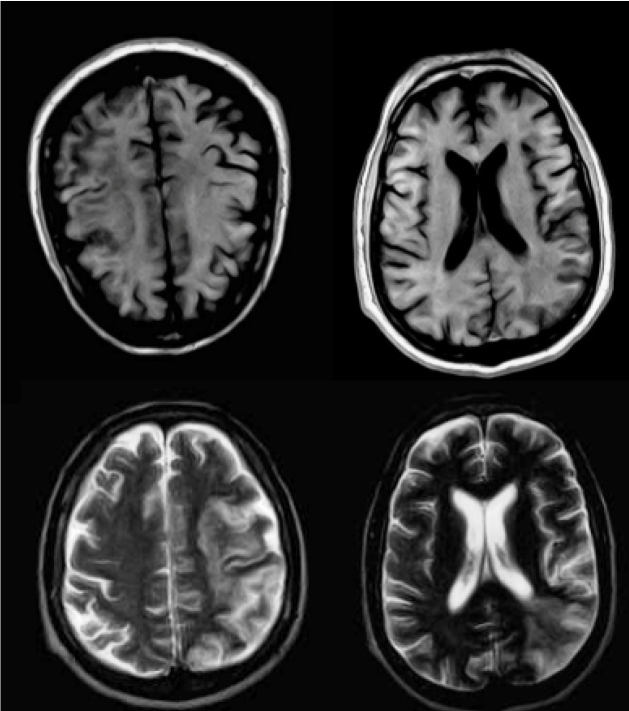
Magnetic resonance imaging of the skull in T1 showing hyposignal in the territory of the artery (left cerebral artery), with high intrinsic cortical signal, and hyperintensity in the T2 sequence.

A T1-weighted magnetic resonance sequence showed an area of hypointensity, with a high intrinsic cortical signal, and hyperintensity in the T2 sequence (Figure 2). A Doppler velocimetric study of the left carotid artery determined total/critical sub occlusion. These findings corroborated the clinical diagnosis of subacute ischemic stroke, with an estimated duration of 15 days.

Laboratory tests were also conducted for D-dimer and C-reactive protein (CRP) levels in addition to CSF analysis. These showed no changes (D-Dimer 276 ng/dL and C-reactive protein <6.5). Clinical management followed the institutional protocol with double antiplatelet therapy and atheromatous plaque stabilization therapy with atorvastatin, with no need for admission to the intensive care unit. A multidisciplinary team (including a physiotherapist and speech therapists) monitored the patient on a daily basis, who was eventually discharged with significant neurological improvement.

## DISCUSSION

People living with HIV (PLHIV) are at an increased risk of cardiovascular events. including ischemic strokes, during periods of high viremia or immunosuppression. However, it is not clear whether PLHIV with undetectable viral loads and good immunity differ in stroke incidence compared to people without HIV^2^.

In addition to the traditional risk factors, HIV is associated with other factors that increase the risk of stroke, including high viremia, immunosuppression, and the use of antiretrovirals such as protease inhibitors, which can lead to dyslipidemia and lipodystrophy (e.g., lopinavir and indinavir). Furthermore, a previous meta-analysis showed that patients who used abacavir were at an increased risk of cardiovascular disease, with those of advanced age likely to have better immunity control and more prevalent traditional risks^2^. 

The etiology of stroke in PLHIV is multifactorial, including atherosclerosis of the large arteries, small vessel disease, cardio embolism, central nervous system (CNS) infections, coagulation disorders, and non-atherosclerotic vasculopathy. At the initial assessment of PLHIV and ischemic stroke, it is advised to assess immunological status, which can direct the investigator to some probable etiologies. However, the triggering mechanism for stroke remains unclear, which makes it difficult to manage the condition among members of this population^2^.

It is unclear how SARS-CoV-2 specifically affects PLHIV. In 2003, research conducted in the Middle East showed that PLHIV were at a lower risk of serious infection from SARS-CoV, which may have resulted from the correct use of antiretroviral therapies. However, they also tended to experience more prolonged illnesses due to immunosuppression from HIV infection^3^. 

It was not possible to relate the severity of the condition presented with other associated comorbidities, nor in regard to the TCD4 lymphocyte count or relationship between CD4/CD8. This does not support the idea that PLHIV experience more severe courses of SARS-CoV-2 than others. It is not possible to relate infection by the HIV virus with a greater susceptibility to infection by SARS-CoV-2 or more severe conditions of the disease^3^. 

Several pathophysiologies have been proposed to explain the neurological involvement of patients with confirmed cases of SARS-CoV-2, including direct injury, hypoxic injury, immunological injury, and neuroinvasion through receptors of the angiotensin-converting enzyme 2 (ACE2). This involvement has no direct relationship with either the onset of pulmonary involvement or its severity^4^. 

SARS-CoV and SARS-CoV-2 are considered to be predominantly respiratory viruses. In this context, a common clinical manifestation is hypoxia, which can lead to cerebral edema resulting in varied neurological manifestations. However, these viruses can directly infect neural cells, manifesting with encephalopathy, loss of visual acuity, and cerebral herniation. Previous studies have already reported patients with respiratory failure due to brainstem involvement^5^ ^,^ ^6^. 

The pathophysiology of stroke in patients with confirmed SARS-CoV-2 is not yet well defined. However, inflammatory activity resulting from the infection, with activation of the interleukin cascade, can result in coagulation abnormalities, which contribute to the development of thromboembolic events. Under these conditions, it is necessary to identify patients with a high potential for thromboembolic events through laboratory monitoring and dosages of inflammatory markers (CRP and D-dimer)^7^. 
